# Patient experiences and needs in cancer care– results from a nationwide cross-sectional study in Germany

**DOI:** 10.1186/s12913-024-10951-y

**Published:** 2024-05-02

**Authors:** Elâ Ziegler, Jens Klein, Christopher Kofahl

**Affiliations:** Institute of Medical Sociology, University Medical Center Hamburg-Eppendorf, Martinistraße 52, 20246 Hamburg, Germany

**Keywords:** Cancer care, Oncology, Patient satisfaction, Patients’ needs, Patient experience, Physician-patient interaction, Patient-centred care, Patient-reported outcomes

## Abstract

**Background:**

Patient-centredness has become a central quality indicator for oncology care. Elements include shared decision-making, patient navigation and integration of psychosocial care, which impact patient-reported and clinical outcomes. Despite efforts to promote patient-centred care in Germany in recent decades, implementation remains fragmented. Further, research on patient experiences with cancer care and its determinants is limited. Therefore, this study examines which patient- and facility-specific factors are associated with patient-centred quality care delivery.

**Methods:**

A cross-sectional study was conducted among 1,121 cancer patients in acute treatment, rehabilitation, and aftercare for different cancer entities across Germany. A participatory developed questionnaire was used. Outcome measures were the quality of physician-patient interaction and provision of psychosocial care during acute care. Predictors comprised patient-specific characteristics and treatment facility-specific factors. Multiple linear regression and multivariate binary logistic regression analyses were performed. In addition, a content analysis of open-ended comments on the patients’ overall cancer care needs was applied.

**Results:**

Multiple linear regression analysis showed recent diagnosis (β=−0.12, *p* = < 0.001), being male (β=−0.11, *p* = 0.003), and having a preference for passive decision-making (β=−0.10, *p* = 0.001) to be significantly associated with higher interaction quality, but not age, education and health insurance type. An overall low impact of patient characteristics on interaction quality was revealed (adj. R^2^ = 0.03). Binary logistic regression analysis demonstrated the availability of central contact persons (OR = 3.10, *p* < 0.001) followed by recent diagnosis (*p* < 0.001), having breast cancer (*p* < 0.001) and being female (OR = 1.68, *p* < 0.05) to significantly predict offering psycho-oncological counselling to patients in acute care facilities. The availability of peer support visiting services (OR = 7.17, *p* < 0.001) and central contact persons (OR = 1.87, *p* < 0.001) in the care facility, breast cancer diagnosis (*p* < 0.001) and a higher level of education (*p* < 0.05) significantly increased the odds of patients receiving information about peer support in the treatment facility. Despite relatively satisfactory quality of physician-patient interactions in cancer care (M = 3.5 (± 1.1)), many patients expressed that better patient-centred communication and coordinated, comprehensive cancer care are needed.

**Conclusion:**

The findings reflect effective developments and improvements in cancer care and suggest that patients’ social characteristics are less decisive for delivering patient-centred quality care than systemic factors surrounding the care facilities. They can serve to inform oncology care in Germany.

**Supplementary Information:**

The online version contains supplementary material available at 10.1186/s12913-024-10951-y.

## Background

Patient-centred care and the measurement of its quality through patient-reported outcome measures (PROMs) and patient-reported experience measures (PREMs) have gained relevance in the field of oncology over the past decades [1, 2]. Since research has shown that patient-oriented approaches to health care can potentially improve chronic patients’ care experiences and clinical outcomes [3–5], it has become a key component of quality care [1, 4, 6]. Subsequently, patient-centred cancer care has been promoted through various initiatives [7].

In Germany, the Federal Ministry of Health initiated a National Cancer Control Plan (NCCP) in 2008 to provide high-quality, comprehensive care and to strengthen patient-centredness in cancer care comprising acute care, rehabilitation and aftercare [8]. It was driven by the German Cancer Society, which also developed other initiatives to foster excellent patient-centred care, such as the certification system for cancer centres, as one part of the NCCP [9, 10]. Further efforts are reflected in disease management programmes for cancer patients [11] anchored in German health regulations and in the concept of self-help-friendliness [12]. Those enable patient-centred and integrated care that considers supportive psychosocial cancer care (e.g. peer support). In particular, the importance of successful patient communication and shared decision-making (SDM) is increasingly recognised as an integral feature of patient-centred care [13–15]. Research has emphasised the significance of high-quality physician-patient relationships, with SDM being an important element for treatment and adherence [16, 17]. These relationships have also been found to correlate with patient satisfaction and improved decision-making in cancer care [18]. Therefore, several supportive tools and training programmes for healthcare professionals have been introduced in Germany [19, 20]. Other examples of changes in German cancer care are patient involvement in multidisciplinary tumour boards [21] and progress in patient navigation [22]. The latter has led to the provision of oncology nurse navigators or similar contact persons in some cancer facilities across Germany [23, 24]. This, in turn, has been shown to positively influence patients’ satisfaction with care and relates to their quality of life [22, 25].

Although these developments are encouraging and promising, they have not been systematically implemented in all cancer care facilities [26]. Initiatives remain scattered, as there is no standard for establishing them in Germany to target patient-centred care, mainly due to systemic reasons surrounding healthcare [15, 27]. In addition, other framework conditions affect patient-centred quality care. For instance, oncological care units are exposed to economic constraints and staff shortages while patient numbers increase [28]. This trend results in less time available for patients and thus can lower physician-patient interaction quality [29] and hinder cooperation with psychosocial cancer care [30]. The introduction of diagnosis-related groups (DRGs) has exacerbated these developments and may decrease the quality of care experienced by patients [31]. Moreover, the COVID-19 pandemic negatively impacted and limited cancer care, particularly concerning psycho-oncological care [32].

Recent data on patient satisfaction with care revealed positive experiences of many cancer patients in Europe [33–35] but also demonstrated persisting unmet needs concerning communication and coordinated supportive psychosocial care [34–36]. Despite the shift towards patient-centred care and numerous changes within the oncology care system, studies suggest that the quality of cancer care in some European countries is still determined by patient characteristics such as age, educational background and time to treatment [34, 37–39]. For Germany, current data assessing patient satisfaction and determinants of quality cancer care is limited. Thus, this study aims to gather the care experiences and needs of cancer patients in Germany and to assess satisfaction with care to identify strengths and weaknesses of oncological care and its developments. In particular, the goal of this study was to examine which patient-related and facility-specific factors correlate with different indicators of patient-centred care within the German oncology care system to inform it further. Subsequently, the objectives of this research are: (1) to examine which patient- and cancer care facility-specific factors are associated with better physician-patient interaction and (2) are associated with the offering of psychosocial support in acute care, and (3) to identify the overall cancer care needs of patients.

## Methods

### Study design, population and recruitment

This study applied a cross-sectional design. Data were collected between October 2020 and September 2021 with a self-administered questionnaire to investigate care experiences and needs of cancer patients in Germany. The survey is part of a project examining health literacy and peer support activities of cancer patients. It follows a participatory research approach and has been conducted in cooperation with the House of Cancer Self-Help– Federal Association (HKSH), an association of ten nationwide cancer peer support organisations (PSOs). Ethical approval was granted by the Local Psychological Ethics Committee at the Center for Psychosocial Medicine of the University Medical Center Hamburg (No. LPEK-0109).

German-speaking participants and non-participants of peer support groups (PSGs) aged 18 years and older with a cancer diagnosis of any type and at any stage, were eligible to participate. A multi-channel approach was adopted for broad patient recruitment. More than 60,000 brochures and posters with information about the study were sent to 1,382 cancer care providers for acute care, rehabilitation, aftercare and supportive psychosocial care throughout Germany from October 2020 onwards. These care and counselling facilities, such as cancer societies, cancer counselling centres, oncological rehabilitation clinics, certified cancer centres, hospitals with oncological departments, and oncological specialised practices and PSOs, were notified of the study in advance by mail. Snowball sampling of patients also took place through the HKSH, peer support groups outside PSOs, and the German Cancer Society to disseminate the call for study participation (via email, post and PSO journals). In addition, the study information was circulated in a newsletter of the National Contact and Information Centre for the Initiation and Support of Self-Help Groups (NAKOS) and at an online patient congress. Reminder emails were sent in February and May 2021 to increase the response rate. Patients could choose between online and paper-pencil participation. Participation was anonymous, and all participants gave their informed consent by confirming (online or by sending in the paper questionnaire) that they had read the study information and data protection policy, and that they are willing to participate.

### Measures and variables

The questionnaire used in this research is part of a larger survey and covers various aspects such as diagnosis and treatment, care experience, socio-demographics, and other dimensions (see supplementary file 1). It was newly developed in collaboration with representatives of oncology, medical sociology, PSOs and PSGs and is based on a previous data collection that included qualitative interviews and a quantitative survey with PSG leaders, as well as a literature screening of instruments.

#### Outcome variables

A modified version of the 14-item Questionnaire on the Quality of Physician-Patient Interaction (QQPPI) [40] was used to measure patients’ perception of the quality of physician-patient interaction in consultations during acute cancer care. The QQPPI was validated in a sample of patients in outpatient care and showed very good internal consistency (Cronbach’s α = 0.95) [40]. We used 7 items of the original QQPPI scale (items 2, 4, 6, 7, 8, 11, 14). The other items were deleted to make the questionnaire generalisable to any type of cancer care settings, in line with the authors’ recommendation to use a shortened version [40]. Two new items (numbers 8 and 9) were added as they were identified by cancer PSO members as important to patients. The 9-item scale showed identical reliability (Cronbach’s α = 0.95). The scale items refer to statements about the interaction and are to be rated on a 5-point Likert scale ranging from 1 ‘does not apply’ to 5 ‘fully applies’ (see supplementary file 2). For analyses, a sum score ranging from 1 (low quality) to 5 (high quality) was calculated from the 9 items. Accordingly, successful interaction of high-rated quality is characterised by detailed information from the physician regarding treatment and illness, sufficient time, taking the patient seriously, and involving the patient and their relatives in treatment decisions.

Two binary outcome variables as indicators of quality care assessed the provision of psychosocial services in the acute treatment facility. One item asked whether the patients were offered psycho-oncological counselling (see supplementary file 1, item B.5a), and another whether they received information about the possibility of participating in a PSG (item C.2) during acute care (each with the response option 0 = no or 1 = yes).

In addition, in the sense of patient involvement and to obtain more detail on patients’ experiences and opinions, a content analysis was conducted on open-ended responses to the question of whether the patients had any specific wishes and needs regarding their overall cancer care (see supplementary file 1, item B.10). As the responses were provided as bullet points or short sentences, they were grouped and categorised inductively into broad themes by two researchers using Excel spreadsheets, applying a simple qualitative content analysis [41] to provide (frequency) counts through content coding.

#### Independent variables

Patient-related predictors comprised social (sex, age, education), clinical (time since diagnosis, cancer type) and healthcare-specific characteristics (insurance type, decision-making style). Sex was dichotomised in 0 = male and 1 = female. Age (at diagnosis) was coded as a continuous variable in years by subtracting years since diagnosis from age at time of participation. Schooling was coded into low (= 1), medium (= 2) and high (= 3), i.e. ≤ 9 years of education; 10 years of education and ≥ 11 years of schooling and takes into account the three different schooling types in Germany. Time since diagnosis was grouped into the following three categories: 1 ‘<1 year (newly diagnosed)’, 2 ‘diagnosis 1 to < 5 years’ and 3 ‘5 + years (cancer survivors)’. Type of self-reported primary cancer diagnosis was grouped based on distribution into breast cancer (= 1), prostate cancer (= 2) and other cancer types (= 3). Besides, the type of health insurance was assessed and dichotomised into 1 = statutory and 2 = private health insurance. To measure patients’ preference regarding medical decision-making, a modified version of item 13 of the Patient Participation Questionnaire [42] was used to assess participants’ general attitudes towards different models of decision-making. The wording in the question was changed from “Who should make the medical decisions about your problems” to “Who in your opinion should make the medical decisions for the diseases you have?” and gendered the response options (see item E.10 in the supplementary file 1). Patients were asked to indicate who should make the medical decisions for their disease, with values ranging from 1 = passive (equivalent to paternalistic model preference; ‘The doctor should decide’) to 5 = active (corresponding to informed decision-making model preference; ‘I should decide’). For binomial logistic regression, patients’ preference for medical decision-making was grouped into three categories in analogy to the models of physician-patient relationships (1 = paternalistic model; 2 = SDM, 3 = informed decision making).

Predictors relating to care facility characteristics were examined through two single items. One item assessed if there was a central contact person available in the hospital to guide the patients through treatment and further care, and a second item assessed whether visiting services of PSGs were available in the hospital (response options 0 = no, 1 = yes).

### Statistical analyses

Data analysis was performed using IBM SPSS Statistics 27. Descriptive statistics were used to examine the participants’ clinical and socio-demographic characteristics and the distribution of the values of the outcome variables. Missing value imputation by using mean value imputation and series mean imputation were performed for missing single item data. If crucial data on sociodemographic information or outcome variables was missing, cases were excluded from analyses. For scales, a maximum of three missing responses were tolerated. If more than three items were missing, the total score was treated as a missing value. Multiple linear regression was used to assess the quality of the interaction considering patient-specific variables described above. The method of regression was forced entry and cases were excluded listwise. Multivariate binary logistic regression analyses were conducted to assess the associations between multiple patient- and facility-specific variables, and the provision of psychosocial care in acute cancer care. The method of logistic regression was forced entry, with all variables being entered into the models and the values of the relevant parameters were estimated using maximum-likelihood estimation. Reference categories were set as the first. Statistical significance was set at an α level of 0.05 for all analyses.

## Results

### Patient characteristics

A total of 1,356 patients responded to the study. Respondents with missing information on socio-demographic characteristics were excluded. After data cleaning, the dataset consisted of 1,121 participants who completed the questionnaire, mainly online (84%). The patients’ characteristics are shown in Table 1. Patients from all 16 federal states participated, the majority of them from North Rhine-Westphalia, the most populous state in Germany. Both newly diagnosed patients and cancer survivors participated, with an average time of 4.6 ± 6.0 years after cancer diagnosis. About 30% of patients were still undergoing treatment at the time of the survey. The participants’ mean age was 61.3 ± 12.4 years at participation. Most participants were female (54.7%), had a high level of education (58.3%) and lived in a partnership (83.2%). The respondents were predominantly breast cancer patients (30.6%), followed by prostate cancer patients (19.3%). Cancer stages varied, ranging from UICC (Union Internationale Contre le Cancer) stage 0 to IV, with most patients not knowing their disease stage (43.5%). Less than half of the participants were members of PSGs (45.2%). Most patients preferred SDM (76%) and had statutory health insurance (83.1%).

**Table 1:** Characteristics of the patients (*N* = 1,121)

Patient characteristic	n (%) or Mean (SD)
Age (years)	61.3 (± 12.4)
Sex	
Male	507 (45.3%)
Female	613 (54.7%)
Educational level	
Low	135 (12.2%)
Medium	326 (29.5%)
High	645 (58.3%)
Partnership	
No	184 (16.8)
Yes	909 (83.2%)
Primary cancer type	
Breast cancer	337 (30.6%)
Prostate cancer	212 (19.3%)
Bladder cancer	91 (8.3%)
Colorectal cancer	77 (7.0%)
Other (overall each less than 5%)	383 (35.3%)
UICC stage	
0	16 (1.5%)
I	114 (11.0%)
II	160 (15.4%)
III	210 (20.3%)
IV	86 (8.3%)
Do not know	451 (43.5%)
Time since diagnosis (years)	4.6 (± 6.0)
< 1	345 (30.9%)
1 to < 5	370 (33.1%)
5+	403 (36.0%)
Decision making preference	
Paternalistic model	69 (6.2%)
Shared decision-making	841 (76.0%)
Informed decision-making	197 (17.8%)
Health insurance	
Statutory	919 (83.1%)
Private	187 (16.9%)

Abbreviation: UICC, Union Internationale Contre le Cancer

### Quality of physician-patient interaction

On a scale from 1 (low) to 5 (high), the average quality score for physician-patient interaction during consultations in acute cancer care was 3.5 (± 1.1) among respondents. To determine the association between the quality of physician-patient interaction in care facilities and patient characteristics, a multiple linear regression was performed. Before regression analysis, normality, linearity, multicollinearity, and independence of residuals were checked and no concerns were found. Six explanatory variables were entered into the regression model with the quality of physician-patient interaction as the outcome variable (Table 2). Indicating a normal distribution, the analysis revealed the model to be a poor model fit to the data (F(6,1010) = 5.43, *p* < 0.001), being statistically significant and explaining 3% of the variability in the dependent variable (adjusted R^2^ = 0.03). The results revealed more recent diagnoses (β=−0.12, *p* = < 0.001), being male (β=−0.11, *p* = 0.003) and patients’ preference for a more passive role in decision-making (β=−0.10, *p* = 0.001) to be significantly associated with interactions of better quality.

**Table 2:** Linear regression model examining quality of physician-patient interaction (*n* = 1,017)

Independent variables	Regression coefficient Β	95%-CI	Standard error	Standardised regression coefficient β	p
Age at diagnosis	-0.001	-0.01-0.01	0.003	-0.016	0.648
Sex (male = 0, female = 1)	-0.234	-0.39–0.08	0.078	-0.107	**0.003**
Education	0.026	-0.07-0.13	0.051	0.017	0.608
Years since diagnosis	-0.155	-0.24–0.07	0.044	-0.115	**< 0.001**
Decision making preference	-0.175	-0.28–0.07	0.054	-0.102	**0.001**
Health insurance (statutory = 0, private = 1)	0.135	-0.05-0.31	0.092	0.047	0.142

Significant variables highlighted in bold

Adjusted R^2^ = 0.03

### Other quality care indicators

#### Provision of psycho-oncological counselling

In total, 55.5% (*n* = 587) of respondents were offered psycho-oncological counselling in the hospital for acute treatment. The majority of them (63.4%) made use of this offer. To test which patient- and facility-specific factors shape whether cancer patients were offered psycho-oncological counselling or not, a binomial logistic regression was conducted. All model coefficients and odds can be found in Table 3. The model was statistically significant (χ²(12) = 203.67, *p* < 0.001). Goodness-of-fit was assessed using the Hosmer-Lemeshow-Test, indicating a good model fit, χ²(8) = 4.78, *p* > 0.05. Of the eight variables entered into the regression model, four contributed significantly to predicting the provision of psycho-oncological counselling: sex (*p* < 0.05), cancer type (*p* < 0.001), time since diagnosis (*p* < 0.001) and availability of a central contact person in the hospital (*p* < 0.001), while the other variables showed no significant associations. Being female increased the likelihood of provision of psycho-oncological support, OR = 1.68 (95%-CI[1.13, 2.50]), and the availability of a central contact person for patients, OR = 3.10 (95%-CI[2.19, 4.39]), as patients had 3.1 times higher odds of being offered psycho-oncological counselling if a central contact person was available in the hospital. Having a cancer diagnosis other than breast cancer (e.g. prostate cancer OR = 0.32 (95%-CI[0.18, 0.57]), and a diagnosis longer ago ( 5+ years) compared to patients with a recent diagnosis (< 1 year), OR = 0.42 (95%-CI[0.29, 0.62]) decreased the likelihood of psycho-oncological support provision.

**Table 3:** Logistic regression model predicting offer of psycho-oncological counselling (*n* = 967)

Independent variables	OR	95%-CI	p
Age at diagnosis	0.99	0.97-1.00	0.052
Sex (Reference: Male)	1.68	1.13–2.50	**0.010**
Education			
Low	1.00		
Medium	1.11	0.67–1.85	0.686
High	1.02	0.63–1.65	0.942
Primary cancer type			
Breast cancer	1.00		
Prostate cancer	0.32	0.18–0.57	**< 0.001**
Other	0.52	0.35–0.77	**< 0.001**
Years since diagnosis			
< 1	1.00		
1 to < 5	0.72	0.50–1.04	0.076
5+	0.42	0.29–0.62	**< 0.001**
Decision making preference			
Paternalistic model/Passive	1.00		
Shared Decision Making	1.07	0.58–1.97	0.831
Informed Decision Making/Active	1.19	0.60–2.35	0.626
Health insurance (Reference: Statutory)	1.02	0.70–1.49	0.911
Central contact person available (Reference: No)	3.10	2.19–4.39	**< 0.001**

All variables entered into the model

Significant variables highlighted in bold

Nagelkerke’s R²=0.254

#### Information about peer support participation

Of all respondents, 33.3% (*n* = 327 patients) were informed in the hospital about cancer PSGs. A second multivariate binomial logistic regression examined what factors determine the provision of this information about PSGs and the possibility of participating in them. Nine variables were entered into the final regression model. The model was statistically significant (χ²(13) = 155.19, *p* < 0.001) and showed a good model fit, χ²(8) = 9.18, *p* > 0.05. As presented in Table 4, four of the nine variables entered into the regression model contributed significantly to predicting the provision of PSG information. In particular, if visiting services of PSGs were available in the hospital compared to non-availability, patients were 7.2 times more likely to have been informed about the possibility of participating in a PSG (OR = 7.17 (95%-CI[4.75, 10.82]), *p* < 0.001). Similarly, the availability of a central contact person for patients, OR = 1.87 (95%-CI[1.33, 2.65]) significantly increased the likelihood of provision of information about PSGs (*p* < 0.001). Besides, breast cancer patients compared to prostate cancer (OR = 0.51 (95%-CI[0.27, 0.97]), *p* < 0.05) or other entities (OR = 0.41 (95%-CI[0.27, 0.64]), *p* < 0.001), and patients with medium educational levels compared to low education, OR = 1.78 (95%-CI[1.00, 3.17]), were more likely to have been provided PSG information than not, while other patient-related variables were non-significant.

**Table 4:** Logistic regression model predicting receiving information about peer support (*n* = 864)

Independent variables	OR	95%-CI	p
Age at diagnosis	0.99	0.98–1.10	0.391
Sex (Reference: Male)	0.79	0.49–1.25	0.321
Education			
Low	1.00		
Medium	1.78	1.00-3.17	**0.050**
High	1.70	0.98–2.94	0.060
Primary cancer type			
Breast cancer	1.00		
Prostate cancer	0.51	0.27–0.97	**0.041**
Other	0.41	0.27–0.64	**< 0.001**
Years since diagnosis			
< 1	1.00		
1 to < 5	0.97	0.66–1.45	0.895
5+	1.07	0.70–1.62	0.767
Decision making preference			
Paternalistic model/Passive	1.00		
Shared Decision Making	1.23	0.63–2.38	0.548
Informed Decision Making/Active	1.10	0.52–2.33	0.807
Health insurance (Reference: Statutory)	0.90	0.60–1.38	0.639
Central contact person available (Reference: No)	1.87	1.33–2.65	**< 0.001**
Visiting services available (Reference: No)	7.17	4.75–10.82	**< 0.001**

All variables entered into the model

Significant variables highlighted in bold

Nagelkerke’s R²=0.227

### Patient needs for care

In free text comments, 411 patients stated special wishes and needs regarding their previous oncological care. Those expressed by more than 10 respondents each are listed in Table 5. The most frequently mentioned theme identified relates to more education and information about medical findings, risks, side effects and consequences of treatments (*n* = 137). Another theme was the choice of therapy. In this regard, 125 patients emphasised that they would have preferred other treatment options and that further care options such as “pain treatment”, “incontinence care” or “rehabilitation for follow-up treatment” were missing. Another aspect of patient needs identified concerned better communication between patient and physician, pointed out by 39 respondents. Participants desired conversations at “eye level” with more “empathy”, “respect” and “to be taken seriously”. Other key wishes for patients’ cancer care were categorised into increased social support and greater inclusion of supportive psychosocial care such as psycho-oncological and peer-support services. Here, participants stated that psychosocial services are often not mentioned or recommended, although they are needed both after and during treatment as a kind of “co-care”, which in their view should be offered “at every step of the treatment”.

The above-mentioned categories are framed by patients’ expressed needs relating to certain structures of cancer care. For instance, participants noted the lack of time of the staff in care facilities (*n* = 23). Subsequently, some respondents called for improved framework conditions in hospitals (*n* = 13). Other mentions referred to improved aftercare (*n* = 28) that is needed as “there are significant gaps in care and counselling” and cooperation between physicians, clinics and rehabilitation facilities was lacking. One patient affirmed in this context: “Those affected want structured aftercare with a specialised aftercare and treatment concept”. A stronger involvement of relatives was another need (*n* = 13). Some patients (*n* = 29) also asked for a central, accessible contact person, such as oncology navigators or at least “always the same doctor” responsible for the patients and who can be contacted “in case questions arise or there is a need for information, and who is also familiar with the case”.

**Table 5:** Patients’ needs for cancer care identified from open-ended responses (*n* = 411)

Need/Wish	n	Exemplary responses
More education and treatment information	137	*“Better information overall”*
Better choice of comprehensive therapy	125	*“Rehabilitation for follow-up treatment”.*
Improved patient-oriented physician-patient communication	39	*“Patient-oriented communication; the conversations were often demoralising”.*
Greater social support	33	*“Support by social services (did not take place! )”*
Greater inclusion of psycho-oncology and peer support services	32	*“Psycho-oncological-psychosomatic treatment”*
Central, accessible contact persons (doctors, onco-navigators)	29	*“I would have liked to have an (onco-)navigator as a fixed contact person”*
Improved (coordinated) aftercare	28	*“Structured aftercare with a specialised aftercare and treatment concept is what those affected want”*
More time of practitioners	23	*“The time pressure of medical staff is generally too great”*
Greater involvement of relatives	13	*“Direct information is better than information about the patient”*
Improved operating conditions of care facilities	13	*“In my opinion, there is a fault in the system…”*

## Discussion

The findings propose that contrary to previous data [34, 37–39], the quality of physician-patient interaction is not so much determined by the social characteristics of the patients. This is indicated by the non-significant results for the variables age and education alongside insurance type. Therefore, the findings do not support previous results suggesting that older patients [34, 38, 39, 43] and patients with higher educational levels [37, 43] experience better interactions in cancer care. As a more recent time of diagnosis was the most decisive factor for a better quality of the physician-patient interaction and the R^2^ of the linear regression model was relatively small, the results suggest that its quality is rather determined by other system- and facility-related factors of cancer care not assessed in the study. These might be aspects addressed in the free text comments, such as staff and time available in the care facility. Overall, these results are encouraging and may reflect successful changes in oncology care towards improved patient-centred care. Interestingly, male and more passive patients regarding decision-making showed significantly better interaction quality, albeit with a small effect. Possibly, these patients are less critical in reviewing their experience and more trusting in the assessment of their physicians and their overall care, raise fewer questions about their disease and treatment and are less likely to talk about comprehensive needs [44]. Similarly, Heerdegen et al. [38] demonstrated that male patients were more likely to experience ‘excellent’ cancer care.

Consistent with previous literature [18, 33–35], the assessed quality of physician-patient interaction correlates highly with overall patient satisfaction with care, highlighting its importance and showing that patients are relatively satisfied with the quality in total, as participants scored an average of 3.5 points out of 5 on the interaction quality. Considering that more than a third of the patients were diagnosed and treated during the COVID-19 pandemic, the results possibly indicate the provision of continued quality cancer care despite pandemic-related difficulties [32]. This is confirmed by an additional data analysis, which showed that less than 10% of these participants experienced any treatment changes or cancellations due to the pandemic (data not shown). However, the scores on interaction quality varied widely on a few items, indicating some potential for improvement. This is supported by the identified patients’ needs from the free text comments concerning better patient-centred communication and interaction. Previous studies have also shown that communication is often an unmet need [34, 35], and that poor physician-patient communication can be a barrier to SDM [45], which should be implemented more often [15, 26, 27].

Regarding other quality care indicators, the results further support the finding that instead of patient characteristics, systemic improvements in cancer care are more relevant in determining delivery of patient-centred care, such as providing supportive psychosocial offers. In particular, the availability of central contact persons in the hospital e.g. oncology nurse navigators, significantly increased the likelihood of offering psycho-oncologic counselling and was found to be more likely for patients with a more recent diagnosis. Moreover, as breast cancer patients, compared to other cancer diagnoses, showed a higher likelihood of having been provided psycho-oncologic support, this could also indicate the successful implementation of integrated care for breast cancer, driven by disease management programmes and establishing breast care nurses [11]. As breast cancer patients in Germany are often treated in certified cancer centres, this may also reflect the effect of certification systems by the German Cancer Society [9, 10] to promote integrated cancer care. Additionally, independent from cancer type, female patients were found to be more likely to receive psycho-oncologic counselling, yet with a lower impact. This might stem from the assumption that female patients often have higher levels of distress and hence higher needs but are also more willing to participate in psychosocial care and more likely to recognise their need for such than men [44]. Although not significant but by trend, younger age at diagnosis was associated with higher likelihood of psycho-oncological counselling provision. To explain this, physicians may expect higher distress in younger patients, as cancer incidences are overall lower, and diagnoses can potentially be more drastic among younger age groups [44]. These findings are in line with previous studies showing that physicians judge patients’ needs for psychosocial services based on their sex and age [35, 44].

Central contact persons were also crucial for the provision of information about PSGs in cancer treatment facilities, in addition to PSG visiting services, while patient-related characteristics other than higher education and type of cancer did not prove to be relevant determinants. While this may not seem surprising, it demonstrates that the one measure of patient-centeredness is a precondition for patient-oriented, integrated care, or propels its next level. The result underlines the importance of central contact persons acting as patient navigators and promoters of psychosocial services in cancer care. As breast cancer patients were more likely to receive information about PSGs, the structured, comprehensive care approach to breast cancer care appears to enhance both the provision of professional counselling and peer support as forms of psycho-oncological support. Further, it can be assumed that integrated care benefits from established patient organisations, such as Germany’s largest cancer PSO, Frauenselbsthilfe Krebs, which is particularly committed to supporting women with breast cancer and gynaecological cancers. Other authors have already revealed that delivery of integrated care also positively affects timely access to care and patient-reported satisfaction with care [23, 33, 46]. At the same time, respondents’ free text comments highlight that supportive services are not always sufficiently integrated into cancer care and patients’ wishes for such, as previously mentioned in international literature [30, 34–36]. Yet, the logistic regression models explained only a relatively small amount of variation in the provision of psychosocial care proposing that other factors not depicted here are more influential. These could be disease-related clinical, or facility-related factors, such as the general amount of time available for patients, or physicians’ individual attitudes towards psychosocial care. For instance, not all physicians have a positive attitude towards PSGs and therefore may not suggest them to patients, regardless of their characteristics [30].

Moreover, patients criticise the framework conditions of the hospitals, noting shortcomings in the care system as a whole and aspects of consultations that stem from insufficient time or staff, in line with Osborn’s [28] findings. As a possible solution to some of the issues and needs identified, many patients wished for a central, accessible contact person, such as an oncology nurse navigator. As the provision of such has shown to be positively associated with the quality indicators of patient-centred care, they should be implemented more consistently across cancer care facilities in Germany. Integrated care can further be supported by making stronger use of concepts such as self-help friendliness in hospitals [12] and enabling PSG visiting services through increased cooperation. Besides, the results represent the need to focus on improving patient-centred comprehensive communication at eye level, which should be considered by physicians and can include SDM. In general, more resources are required to further improve the patient-oriented quality of cancer care.

While the findings are encouraging and map recent developments in cancer care towards more patient-centred care that are noticed by patients, there are several limitations of the study to be considered. Firstly, the current sample is not representative, although a multi-channel recruitment approach was followed to include cancer patients with heterogeneous socio-demographic characteristics. Patients with high educational levels are overrepresented and non-German speaking patient groups with migration backgrounds, who may be particularly vulnerable to experiencing poorer quality of care, are underrepresented. Additionally, more than 40% of the participants were PSG members who potentially review care quality more critical than non-members. Moreover, the participants’ overall satisfaction with their cancer care was high in this study, which could have been different in a more balanced sample. This may have led to a bias in the data towards positive reporting. Besides, the regression analyses mainly focused on patient experiences with acute hospital care and did not cover experiences in rehabilitation and aftercare equally. Due to recruitment during the first peak of the COVID-19 pandemic, fewer patients in inpatient acute care were reached. With the large share of long-term cancer survivors participating, recall bias is likely. Additionally, the study relied on patients’ reported assessments of psychosocial care service provision, which may have further biased the results. Levels of distress, social support and quality of life may have also influenced the provision of psychosocial care, yet those variables were not included in the regression models as they were only reported at the time of study participation and do not necessarily reflect circumstances during treatment immediately after diagnosis. A more balanced sample and the inclusion of further relevant covariates may have increased the explained variance. This should be considered for future research. Lastly, causality cannot be depicted due to the cross-sectional study design.

## Conclusions

Overall, the results show a tendency for cancer patients with a recent diagnosis to experience better patient-centred care and for organisational factors rather than patients’ social characteristics to determine indicators of quality care. Progressive changes and effective strategies to increase patient-centred care, such as disease management programmes and patient navigators, are reflected in the findings. Nevertheless, the results also highlight the need for further implementation and fostering of these approaches across cancer care facilities and cancer entities in Germany. Physicians are encouraged to offer psychosocial support to all patients, regardless of their social characteristics, and to take into account the identified experiences and needs of patients. In particular, physicians are advised to support integrated care through further collaboration with psychosocial care and to improve patient-centred communication at eye level to increase the quality in cancer care delivery.

**Declarations**.

### Electronic supplementary material

Below is the link to the electronic supplementary material.

Supplementary Material 1

Supplementary Material 2

## Data Availability

The datasets used and/or analysed in this study are available from the corresponding author on request.

## Abbreviations

PROMs: Patient-reported outcome measures
PREMs: Patient-reported experience measures
NCCP: National Cancer Control Plan
SDM: Shared decision-making
DRGs: Diagnosis-related groups
HKSH: House of Cancer Self-Help– Federal Association
PSO: Peer support organisation
NAKOS: National Contact and Information Centre for the Initiation and Support of Self-Help Groups
PSG: Peer support group
QQPPI: Quality of Physician-Patient Interaction
UICC: Union Internationale Contre le Cancer
