# Supplementary material for: Patient experiences and needs in cancer care– results from a nationwide cross-sectional study in Germany
Source: BMC Health Serv Res. 2024 May 2;24:572. doi: 10.1186/s12913-024-10951-y (PMC11067160; doi:10.1186/s12913-024-10951-y)
Supplement: Supplementary file 1 — Supplementary Material 1 [file 12913_2024_10951_MOESM1_ESM.docx]

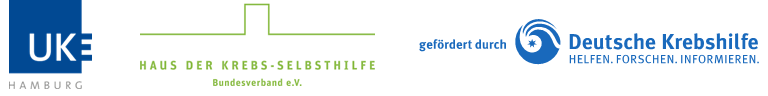


**Questionnaire for people with cancer**

Dear participant,

Thank you very much for taking part in our study gesa-K (health literacy, self-help activities and care experience of people with cancer). The study is funded by the *German Cancer Aid* and carried out in cooperation with the *House of Cancer Self‐Help – Federal Association*. The aim of this study is to gain a better understanding of the living situation and experiences of people with cancer with regard to their illness. We hope that this research will enable us to improve care for people with cancer.

This survey is about your experiences as a cancer patient. The questionnaire on the following pages contains questions about the diagnosis and treatment of your cancer as well as questions about your experiences with your care in hospitals, rehabilitation clinics and your personal coping with the disease. We need this important information in order to obtain a comprehensive picture of the consequences of cancer for those affected and to be able to adapt care to their individual needs.

If a question is less relevant to you or if you find it difficult to decide on an answer, please select the answer that comes to mind spontaneously.

Before the actual survey begins, we would like to know how you found out about this survey and what your current situation is.

| 1 | How did you hear about this survey? (multiple answers possible) | |
| --- | --- | --- |
|  | □ Project website  □ Registered oncologist  □ Registered (organ) specialist  □ Event  □ Self-help group/association  □ House of Cancer Self-Help  □ other, namely:  🖋 | □ National Cancer Society  □ General practitioner  □ Rehabilitation clinic  □ Hospital  □ Other affected persons  □ Cancer Counselling Centre |

| 2 | Where are you currently located? | | |
| --- | --- | --- | --- |
|  | ○ in a rehabilitation clinic | ○ in hospital | ○ at home |

**A - Diagnosis and treatment**

Firstly, we would like to ask you to provide some formal information about your illness, as cancers and their treatments are very diverse. The term **acute treatment** is used several times in the following sections. This refers to the initial cancer treatment (e.g. surgery, temporary drug therapy and radiotherapy).

| 1 | | When were you diagnosed with your current cancer? | | | | / (MM / YYYY) |
| --- | --- | --- | --- | --- | --- | --- |
| 2 | | What type of cancer were you diagnosed with?  🖋 | | | | |
| 3 | | Where did your acute treatment take place? Please state the name and location of the clinic/hospital or practice of your acute treatment. | | Name: __________________________________  Place: __________________________________ | | |
| 4 | | What forms of treatment have you received or used? (multiple answers possible) | | | | |
|  | | □ Watchful Waiting / Active Surveillance  □ Surgery (Operation)  □ Radiotherapy  □ Chemotherapy  □ (Anti-)hormone therapy | | | □ Antibody therapy  □ Homeopathy  □ Complementary medicine procedures  □ other, namely:  🖋  □ don't know | |
| 5  a | | Do you know the staging system of the UICC (Union Internationale Contre le Cancer)?  ○ yes . ○ no | | | | |
| b | | What stage were you diagnosed with? | | | | |
|  | ○ 0 ○ I ○ II ○ III ○ IV ○ don't know | | | | | |
| 6 | | The following list contains common health problems. Please indicate whether you currently have the problem listed. (multiple answers possible) | | | | |
|  | | □ Heart problems  □ Hypertension  □ Lung problems  □ Diabetes/blood sugar  □ Gastrointestinal problems  □ Kidney problems  □ Liver problems | □ Anaemia or other blood problems  □ Depression  □ Osteoarthritis  □ Back pain  □ Rheumatism  □ Other cancers  □ other, namely:  🖋 | | | |

**B - Care experience**

| 1 | Who carried out your acute treatment? | |
| --- | --- | --- |
|  | ○ Doctor at the hospital  ○ Registered oncologist  ○ Registered (organ) specialist | ○ General practicioner  ○ other, namely:  🖋 |

In the following, we would like to ask you for your opinions as a patient on your experiences with your medical care in the context of your **acute treatment.** Please relate your answers to **the clinic**, **practice or hospital where** you were **first** treated for your cancer.

We would now like to know the extent to which you agree or disagree with each statement.

| 2 |  | does not apply | partly applies | applies | strongly applies | fully applies |
| --- | --- | --- | --- | --- | --- | --- |
| a | The doctor informed me in detail about my illness. | ○ | ○ | ○ | ○ | ○ |
| b | The doctor informed me in detail about the available treatment options. | ○ | ○ | ○ | ○ | ○ |
| c | The doctor talked to me in detail about the risks and side effects of the treatment. | ○ | ○ | ○ | ○ | ○ |
| d | The doctor took enough time for me. | ○ | ○ | ○ | ○ | ○ |
| e | The doctor gave me enough opportunities to state my difficulties and problems. | ○ | ○ | ○ | ○ | ○ |
| f | My problems and needs were understood and taken seriously by the doctor. | ○ | ○ | ○ | ○ | ○ |
| g | The doctor determined all treatment measures together with me. | ○ | ○ | ○ | ○ | ○ |
| h | The doctor left the final treatment decision to me. | ○ | ○ | ○ | ○ | ○ |
| i | The doctor encouraged me to involve my relatives in my treatment. | ○ | ○ | ○ | ○ | ○ |

| 3 | | not at all | rather no | partly | rather yes | fully |
| --- | --- | --- | --- | --- | --- | --- |
| a | **During your acute treatment,** did you feel that you were offered sufficient support by the clinic, practice or hospital? | ○ | ○ | ○ | ○ | ○ |
| b | Did you have all the information you needed at the **time of discharge** or onward referral? | ○ | ○ | ○ | ○ | ○ |
| c | Were your wishes taken into account when **planning your further treatment** after discharge or onward referral? | ○ | ○ | ○ | ○ | ○ |

| 4  a | Were the decisions of the **tumour conference** (tumour board) concerning you discussed with you?  ○ yes . ○ no ○ don‘t know |
| --- | --- |

| 5  a | Were you offered **psycho-oncological counselling** in the hospital where you received your acute care?  ○ yes . ○ no ○ don’t know ○ I was not in hospital |
| --- | --- |
| b | *If yes,* have you taken up psycho-oncological counselling?  ○ yes . ○ no |
| c | *If yes,* how helpful did you find the psycho-oncological counselling?  ○ very . ○ quite ○ little ○ not at all |

| 6  a | Was there a **central contact person** (e.g., onco navigator) in the hospital where you received your acute treatment who guided you through your treatment and further care? | | | | |
| --- | --- | --- | --- | --- | --- |
|  | ○ yes | | ○ no | ○ don’t know | ○ I was not in hospital |
| b | | *If yes*, have you obtained information from the contact person?  ○ yes . ○ no | | | |

We consider **relatives and friends of** people with cancer to be among those affected. Therefore, the next questions deal with the situation of your relatives/close ones during your care.

| 7 | | very | fairly | moderately | little | not at all |
| --- | --- | --- | --- | --- | --- | --- |
|  | How important is it to you that your relatives or loved ones are involved in your treatment? | ○ | ○ | ○ | ○ | ○ |

| 8 | | not at all | rather no | partly | rather yes | fully | does not apply |
| --- | --- | --- | --- | --- | --- | --- | --- |
| a | During your acute treatment, did you feel that your **relatives** or **loved ones** were offered sufficient support by the clinic or practice? | ○ | ○ | ○ | ○ | ○ | ○ |
| b | Were the wishes of your **family** or **loved ones** taken into account when planning your continued treatment after discharge or onward placement? | ○ | ○ | ○ | ○ | ○ | ○ |

The following two questions now relate to your **overall** care and no longer just to acute treatment.

| 9 | How satisfied were you overall with your care to date? | | | |
| --- | --- | --- | --- | --- |
|  | ○ very satisfied | ○ satisfied | ○ dissatisfied | ○ very dissatisfied |

| 10 Did you have or do you have any special wishes or needs regarding your previous care? (keywords) |
| --- |
| 🖋 |

**C - Self-help activity**

Many people affected find it helpful to receive support from **self-help groups (SHG)**, for example. We would therefore like to use the following questions to better understand whether and what experiences you have had with self-help offers.

| 1  a | As far as you know, was there a **visiting service of a SHG** in the hospital/clinic?  ○ yes . ○ no ○ don’t know ○ I was not in hospital (continue with question 2) |
| --- | --- |
| b | *If yes,* were you informed in the hospital/clinic about the possibility of a **SHG visiting service**?  ○ yes . ○ no |
| c | *If yes,* have you used the visiting service?  ○ yes . ○ no |

| 2 |  | yes | no | don’t know |
| --- | --- | --- | --- | --- |
|  | Were you or your relatives informed in the hospital/clinic about the possibility of **participating in a SHG** (verbally and/or in writing)? | ○ | ○ | ○ |

| 3 Are you or have you ever been a member of a **cancer self-help organisation** (association or national association)? | |
| --- | --- |
| ○ | no, never |
| ○ | yes, formerly |
| ○ | yes, currently |

| 4 Are you or have you ever been a member of a **cancer self-help group**? | |
| --- | --- |
| ○ | I have *never* been a member of a cancer self-help group. (-> continue with question 6) |
| ○ | I was *formerly* a member of a cancer self-help group from: \|__\|__\|__\|__\| to \|__\|__\|__\|__\| (year) |
| ○ | I am *currently* a member of a cancer self-help group since: \|__\|__\|__\|__\| (year) |

| 5 | If you are or were a member of a cancer SHG: How did you find out about the SHG? | |
| --- | --- | --- |
|  | □ Staff of hospitals or rehabilitation clinics  □ Doctor in private practice  □ Psychotherapist  □ Homepage of the state / federal association  □ (Information) events  □ Flyers and brochures | □ Social media (Facebook, Twitter, Instagram)  □ Family, friends, acquaintances  □ other people affected  □ Cancer counselling centres  □ Self-help clearing houses  □ other, namely:  🖋 |

6 We would like to know your opinion about self-help groups (SHG) in *general*, even if you are not (or were not) a member. To what extent do the following statements apply to you?

|  |  | applies | rather applies | does rather not apply | does not apply | don't know |
| --- | --- | --- | --- | --- | --- | --- |
| a | I don't need a SHG because I have enough other people to talk to. | ○ | ○ | ○ | ○ | ○ |
| b | I think that SHGs are generally very useful and helpful. | ○ | ○ | ○ | ○ | ○ |
| c | Personally, I would feel uncomfortable in an SHG. | ○ | ○ | ○ | ○ | ○ |
| d | I think SHGs do more harm than good. | ○ | ○ | ○ | ○ | ○ |
| e | I’m worried about going to a SHG because it will  make my illness known to others. | ○ | ○ | ○ | ○ | ○ |
| f | I’m afraid that attending a SHG would put a strain on me. | ○ | ○ | ○ | ○ | ○ |
| g | I don't need an SHG because I don't have any problems. | ○ | ○ | ○ | ○ | ○ |
| h | The fates of others would make me despondent. | ○ | ○ | ○ | ○ | ○ |
| i | I will probably join an SHG at a later date. | ○ | ○ | ○ | ○ | ○ |
| j | I could imagine participating in an online SHG. | ○ | ○ | ○ | ○ | ○ |

| 7 Do you have any comments / remarks on the topic of self-help groups? (keywords) |
| --- |
| 🖋 |

**D - Health literacy**

The following section contains a **knowledge test**, i.e. you are asked to answer questions on various topics related to cancer. Some questions are intentionally very difficult, other questions are rather easy. Please do not think too long. Please do not ask anyone else and do not look things up - this would distort the results of the study. If you do not know the answer to a question or are unsure, please select "don't know". A solution sheet with the correct answers will be made available on the project homepage at the end of the data collection with all patients.

| 1 A tumour in stage I means... | |
| --- | --- |
| ○ | small or medium-sized tumours |
| ○ | tumours with metastases |
| ○ | tumours with lymph node involvement |
| ○ | tumours with distant metastases |
| ○ | don't know |

| 2 A drug is effective in 80% of those treated. That is, in how many people does it ***not*** work? | |
| --- | --- |
| ○ | 80 out of 100 |
| ○ | 20 out of 100 |
| ○ | 8 out of 100 |
| ○ | 2 out of 100 |
| ○ | don't know |

| 3 You have read that the incidence of adverse events is 5%. What does that mean? | |
| --- | --- |
| ○ | The majority of people will experience the adverse event. |
| ○ | There is a chance that an adverse event will occur in 5 out of 100 people |
| ○ | An adverse event will occur during 5 out of 100 days. |
| ○ | The severity of the adverse event is 5%. |
| ○ | don't know |

| 4 True or false? Palliative care aims to cure cancer. | |
| --- | --- |
| ○ | true |
| ○ | false |
| ○ | don't know |

| 5 Rebecca was treated for breast cancer (stage II). There is a 10% chance that the cancer will come back in the next 10 years. If Rebecca takes a new drug, this probability is reduced by 30%. In how many out of 100 women taking the drug, like Rebecca, will the breast cancer come back in the next 10 years? | |
| --- | --- |
| ○ | 3 out of 100 women |
| ○ | 7 out of 100 women |
| ○ | 10 out of 100 women |
| ○ | 30 out of 100 women |
| ○ | don't know |

| 6 What is a metastasis? | |
| --- | --- |
| ○ | surgical procedure |
| ○ | scientific analysis |
| ○ | secondary malignant growth |
| ○ | drug |
| ○ | don't know |

| 7 What are cytostatics? | |
| --- | --- |
| ○ | surgical procedures |
| ○ | benign tumours |
| ○ | secondary malignant growth |
| ○ | tumour-active drugs |
| ○ | don't know |

| 8 What is meant by a colonoscopy? An examination... | |
| --- | --- |
| ○ | of the stomach |
| ○ | of the brain |
| ○ | of the blood |
| ○ | of the intestine |
| ○ | don't know |

| 9 Max goes for a cancer screening. This shows a conspicuous finding. However, the subsequent examination shows that Max does not have cancer. What is the term for such an early detection result? | |
| --- | --- |
| ○ | correct positive |
| ○ | false positive |
| ○ | correct negative |
| ○ | false negative |
| ○ | don't know |

| 10 What does the term "adjuvant therapy" mean? | |
| --- | --- |
| ○ | a treatment before the actual intervention (e.g. surgery) |
| ○ | a treatment that directly targets the cancer |
| ○ | a pure chemotherapy without surgery |
| ○ | a further treatment after the primary therapy (e.g. after the removal of a tumour) |
| ○ | don't know |

| 11 When should follow-up rehabilitation or follow-up curative treatment (AHB) for cancer *usually* begin? | |
| --- | --- |
| ○ | after completion of the initial treatment (primary therapy) |
| ○ | after completion of the secondary treatment (secondary therapy) |
| ○ | at the earliest 8 weeks after hospital discharge |
| ○ | at the earliest 6 months after discharge from hospital |
| ○ | don't know |

| 12 What is the maximum period for which people with statutory health insurance are entitled to *sick pay*? | |
| --- | --- |
| ○ | 6 weeks |
| ○ | 24 weeks |
| ○ | 78 weeks |
| ○ | 2 years |
| ○ | don't know |

| 13 Where can I apply for a (severely) disabled person's card? | |
| --- | --- |
| ○ | family doctor |
| ○ | hospital |
| ○ | health Department |
| ○ | pension Office |
| ○ | don't know |

| 14 As a patient, you have the right to ... ? Please tick **all** statements that apply. | |
| --- | --- |
| □ | inspection of the findings (e.g. CT scans, doctor's letters) |
| □ | destruction of your data if you request it |
| □ | free choice of doctor |
| □ | free choice of your therapy |
| □ | a second opinion by another doctor |

**E - Dealing with the disease and self-management**

Everyone has a different need for information and deals with cancer differently. We would like to better understand what channels you may have used to find information about your illness.

| 1 | To what extent did you use **information from the internet** to learn about your cancer? | | | | |
| --- | --- | --- | --- | --- | --- |
|  | ○ almost exclusively | ○ predominantly | ○ rather little | ○ not at all (-> continue with question 4) | |
| 2 | How easy or difficult is it for you to understand information on the internet? | | | | |
|  | ○ very easy | ○ fairly easy | ○ fairly difficult | ○ very difficult |  |
| 3 | How easy or difficult is it for you to distinguish serious from dubious information on the internet? | | | | |
|  | ○ very easy | ○ fairly easy | ○ fairly difficult | ○ very difficult |  |

4 Which of the following sources of information do you know and have you used to **inform** yourself about your cancer and its treatment?

|  |  | I do not know | I know, but I have not used | I know and have used |
| --- | --- | --- | --- | --- |
| a | (Specialist) literature | ○ | ○ | ○ |
| b | Patient guidelines | ○ | ○ | ○ |
| c | Legal texts (e.g. Social Code, Patients' Rights Act) | ○ | ○ | ○ |
| d | Other affected parties | ○ | ○ | ○ |
| e | other, namely: | ○ | ○ | ○ |
| 🖋 |  |  |  |  |

5 Which of the following institutions do you know and have you used to **inform** yourself about your cancer

and its treatment?

|  |  | I do not know | I know, but I have not used | I know and have used |
| --- | --- | --- | --- | --- |
| a | Cancer Counselling Centres | ○ | ○ | ○ |
| b | Cancer Information Service (KID) | ○ | ○ | ○ |
| c | Independent Patient Counselling Service Germany (UPD) | ○ | ○ | ○ |
| d | German Cancer Society | ○ | ○ | ○ |
| e | German Cancer Aid | ○ | ○ | ○ |
| f | Cancer self-help organisation  or group | ○ | ○ | ○ |
| g | other, namely: | ○ | ○ | ○ |
| 🖋 |  |  |  |  |

| 6 Which of the information sources and institutions that you used did you find particularly helpful? |
| --- |
| 🖋 |

7 The following questions relate to how easy/difficult you think it is to deal with health information.

|  | **In your opinion, how easy/difficult is it to ...** | very easy | fairly easy | fairly difficult | very difficult |
| --- | --- | --- | --- | --- | --- |
| a | ...find information about treatments for illnesses that affect you | ○ | ○ | ○ | ○ |
| b | ...to understand what your doctor tells you? | ○ | ○ | ○ | ○ |
| c | ...to judge when to seek a second opinion from another doctor? | ○ | ○ | ○ | ○ |
| d | ...make decisions about your illness based on the information your doctor gives you? | ○ | ○ | ○ | ○ |

| 8 How often do you need **help from other people** to understand medical information (e.g. on instruction leaflets/brochures)? | | | | | |
| --- | --- | --- | --- | --- | --- |
|  | ○ never | ○ rarely | ○ sometimes | ○ often | ○ always |

9 (PAM-13 – not included due to copyright)

| 10 Who in your opinion should make the medical decisions for the diseases you have? | |
| --- | --- |
| ○ | only yourself |
| ○ | mainly yourself |
| ○ | you and your doctor |
| ○ | mainly your doctor |
| ○ | only your doctor |

11 How high do you estimate the **influence of the following aspects on the development of cancer**, i.e. to what extent are they a cause of cancer?

| a | Lifestyle / behaviour: | low | ○ | ○ | ○ | ○ | ○ | ○ | ○ | ○ | ○ | ○ | high |
| --- | --- | --- | --- | --- | --- | --- | --- | --- | --- | --- | --- | --- | --- |
| b | Psyche and personality: | low | ○ | ○ | ○ | ○ | ○ | ○ | ○ | ○ | ○ | ○ | high |
| c | Environmental and work factors: | low | ○ | ○ | ○ | ○ | ○ | ○ | ○ | ○ | ○ | ○ | high |
| d | Social environment: | low | ○ | ○ | ○ | ○ | ○ | ○ | ○ | ○ | ○ | ○ | high |
| e | Hereditary factors: | low | ○ | ○ | ○ | ○ | ○ | ○ | ○ | ○ | ○ | ○ | high |

| 12 Do you have any comments / remarks on dealing with the disease? (keywords) |
| --- |
| 🖋 |

**F - Social support and quality of life**

This section is about the support you receive from your social environment, your relationship with friends, family and acquaintances and various aspects of life.

1-4 (FACT-G – not included due to copyright)

5 Cancer can cause **worries** for many people affected. We would therefore like to ask you to indicate below to what extent the following worries affect you.

|  |  | not at all | a little bit | somewhat | | quite a bit | very much |
| --- | --- | --- | --- | --- | --- | --- | --- |
| a | It scares me that my illness could return after my treatment. | ○ | ○ | | ○ | ○ | ○ |
| b | I am afraid of the further course of the disease. | ○ | ○ | | ○ | ○ | ○ |

| 6 How strongly do you look after your health in general? | | | | | |
| --- | --- | --- | --- | --- | --- |
|  | ○ very strongly | ○ strongly | ○ moderately | ○ less strongly | ○ not at all |

| 7 | How satisfied are you with your health? | | | | |
| --- | --- | --- | --- | --- | --- |
|  | ○ very dissatisfied | ○ dissatisfied | ○ neither | ○ satisfied | ○ very satisfied |

| 8 | How would you rate your overall quality of life? | | | | |
| --- | --- | --- | --- | --- | --- |
|  | ○ very poor | ○ poor | ○ neither | ○ good | ○ very good |

**G - Economic situation**

The following questions relate to your financial situation in connection with your cancer.

| 1  a | Do you have a (severely) disabled person's card? ○ yes ○ no | | | | | | |  |
| --- | --- | --- | --- | --- | --- | --- | --- | --- |
| b | If yes, what degree of disability (GdB) do you have? GdB: Sign (if available): | | | | | | |  |
| 2 | Have you got into financial difficulties because of your cancer? | | | | | | | |
|  | ○ not at all | | ○ a little | ○ moderately | | ○ quite | ○ very | |
| 3 | | What is the average monthly net income of your household as a **whole**? (This refers to the combined income of all household members from work, pension, social assistance, rent, child benefit, long-term care insurance and other sources after deduction of taxes and social security contributions) | | | | | | |
|  | | ○ under 1.000 €  ○ 1,000 € to under 1,500 €  ○ 1,500 € to under 2,000 €  ○ 2,000 € to under 2,500 €  ○ 2,500 € to under 3,000 € | | | ○ 3,000 € to under 3,500 €  ○ 3,500 € to under 4,000 €  ○ 4,000 € to under 4,500 €  ○ 4,500 € to under 5,000 €  ○ 5.000 € and more | | | |

| 4 How high do you estimate the **expenses** for services paid for privately by you in connection with  your illness **in the last 12 months** (e.g. travelling expenses, medical aids etc.)? (in €) |
| --- |
| 🖋 |

| 5 Do you have any comments / remarks about the economic situation? (keywords) |
| --- |
| 🖋 |

**H - Religiosity / Spirituality**

Religiousness and spiritual well-being can influence how people cope with their illness and are often perceived as important by those affected in order to cope better with cancer. Please indicate below which statements about religion and spirituality apply to you.

| 1 | How religious / spiritual would you describe yourself as? | | | | |
| --- | --- | --- | --- | --- | --- |
|  | ○ not at all | ○ little | ○ medium | ○ quite | ○ very |

| 2 | How much does your faith affect the way you deal with the disease? | | | | | |
| --- | --- | --- | --- | --- | --- | --- |
|  | ○ not at all | ○ little | ○ medium | ○ quite | ○ very | ○ does not apply |

| 3 | Was your faith taken into account in the treatment of your cancer? | | | | | |
| --- | --- | --- | --- | --- | --- | --- |
|  | ○ not at all | ○ little | ○ medium | ○ quite | ○ very | ○ does not apply |

| 4 Do you have any remarks / comments on the subject of religiosity / spirituality? (keywords) |
| --- |
| 🖋 |

**I – Corona**

The healthcare system is currently under particular strain due to the spread of the coronavirus and the associated lung disease SARS-CoV-2. The resulting changes in living conditions and medical care can cause additional stress and uncertainty, especially for cancer patients.

| 1 Have you been diagnosed with a coronavirus infection? | |
| --- | --- |
| ○ | no |
| ○ | yes, mild to moderate symptoms, home quarantine |
| ○ | yes, severe symptoms, hospital treatment |
| ○ | yes, very severe symptoms, intensive care treatment |

| 2 Has your treatment(s) been postponed due to COVID-19? | |
| --- | --- |
| ○ | no |
| ○ | yes, because I decided it |
| ○ | yes, because the practitioners decided it |
| ○ | yes, because I and the practitioners decided it |

| 3 Was (were) planned therapy (therapies) exchanged for others due to COVID-19? | |
| --- | --- |
| ○ | no |
| ○ | yes, because I decided it |
| ○ | yes, because the practitioners decided it |
| ○ | yes, because I and the practitioners decided it |

| 4 Have your follow-up appointments been cancelled due to COVID-19? | |
| --- | --- |
| ○ | no |
| ○ | yes, because I decided it |
| ○ | yes, because the practitioners decided it |
| ○ | yes, because I and the practitioners decided it |

| 5 Have psychosocial or psycho-oncological support services been cancelled due to COVID-19? | |
| --- | --- |
| ○ | no |
| ○ | yes, because I decided it |
| ○ | yes, because the practitioners decided it |
| ○ | yes, because I and the practitioners decided it |

| 6 | To what extent do the following statements apply to you? | fully applies | rather applies | does rather not apply | does not apply at all |
| --- | --- | --- | --- | --- | --- |
| a | I am very afraid of a possible (new) infection. | ○ | ○ | ○ | ○ |
| b | I have informed myself in detail about COVID-19 and its risks. | ○ | ○ | ○ | ○ |
| c | I feel at the mercy of the current situation and powerless. | ○ | ○ | ○ | ○ |
| d | I know how to behave in the current situation. | ○ | ○ | ○ | ○ |
| e | I am worried that the quality of my care will deteriorate due to the current situation. | ○ | ○ | ○ | ○ |

| 7 Are there any other aspects that worry you regarding COVID-19? (keywords) |
| --- |
| 🖋 |

**J – Socio-demographics**

Lastly, we would like to ask you a few questions about yourself.

| 1 | What is your gender? | ○ male | ○ female ○ diverse |
| --- | --- | --- | --- |
| 2 | When were you born? | / (MM/YYYY) | |

| 3  a | How many people live permanently in your household, including yourself?  How many people in your household are children under the age of 14? | | Number of people: 🖋  Number of children: 🖋 |
| --- | --- | --- | --- |
| 4  a Are you currently in a steady partnership?  b If yes, does your partner live in the same household? | | ○ yes ○ no  ○ yes ○ no | |

| 5 What is your marital status? | |  | | |
| --- | --- | --- | --- | --- |
|  | ○ Married / registered partnership, living together | | | |
|  | ○ Married / registered partnership, living permanently separated | | | |
|  | ○ single, never been married | | | |
|  | ○ divorced / registered partnership annulled | | | |
|  | ○ Widowed / partner from registered partnership deceased | | | |
| 6 Where do you live? Please enter the first three digits of your postal code: | | | | X X . |
| 7 | What is your highest level of education? | | | |
|  | ○ left without a high school diploma  ○ Sonder-/Haupt-/Volksschulabschluss (lower  secondary school leaving certificate)  ○ Realschulabschluss/Mittlere Reife | | ○ Fachhochschulreife/Abitur  ○ Fachhochschulabschluss/university degree  ○ another educational qualification, namely:  🖋 | |

| 8 | Were you employed **before** your cancer diagnosis? (multiple answers possible) | |
| --- | --- | --- |
|  | If yes:  □ employed  □ freelance/self-employed  □ civil servant  How many hours/week?  🖋  ___________ | If no:  □ on parental leave  □ housewife / husband  □ student  □ old-age pension  □ sick leave/reduced earning capacity pension  □ disability pension  □ unemployed  □ other, namely:  🖋 |
| 9 | What is your current or most recent occupation?  *Please state the exact job title, not the educational qualification or rank.*  🖋 | |

| 10 | What applies to your **current** situation? (multiple answers possible) | | | |
| --- | --- | --- | --- | --- |
|  | □ I am employed  □ vocational reintegration  □ rehabilitation  □ acute treatment  □ I receive sickness benefit | | | □ I receive a reduced earning capacity pension  □ I receive an olg-age pension  □ I receive ALG II (Hartz IV)  □ I live from my own savings  □ other |
| 11 | Has your partner or relative reduced or ended his/her employment due to your cancer? | | | |
|  | ○ no ○ yes, reduced ○ yes, ended | | | |
| 12 How are you covered by health insurance?  ○ statutory ○ statutory with private supplementary insurance ○ private (with/without subsidy)  ○ not at all | | | | |
| 13 | In which country were you born? | | | |
| a  b | ○ Germany  ○ in another country, namely: 🖋 ______________________________________  If you were not born in Germany, in which year did you come to Germany?  🖋 _________ | | | |
| 14 | What citizenship(s) do you have? (multiple answers possible) | | | |
|  | □ German | | □ other, namely: 🖋 | |
| 15 | What country are your parents from? | | | |
|  | Mother: | ○ from Germany  ○ from another country, namely: 🖋 | | |
| 16 | Father: | ○ from Germany  ○ from another country, namely: 🖋 | | |

**Thank you very much for answering the questionnaire!**

Do you have any comments on this survey or any other feedback?

| 🖋 |  |
| --- | --- |
|  |  |
|  |  |
|  |  |
|  |  |

As soon as the study is completed, you will find the results on our project website at www.uke.de/gesa. You can also register for the newsletter here to receive the results of the study by e-mail.
