# Supplementary material for: Patient experiences and needs in cancer care– results from a nationwide cross-sectional study in Germany
Source: BMC Health Serv Res. 2024 May 2;24:572. doi: 10.1186/s12913-024-10951-y (PMC11067160; doi:10.1186/s12913-024-10951-y)
Supplement: Supplementary file 2 — Supplementary Material 2 [file 12913_2024_10951_MOESM2_ESM.docx]

**Supplementary file 2**

Modified 9-item Quality of Physician-Patient Interaction Questionnaire

*Scaling: does not apply – partly applies – applies – strongly applies – fully applies*

1. The doctor informed me in detail about my illness.
2. The doctor informed me in detail about the available treatment options.
3. The doctor talked to me in detail about the risks and side effects of the treatment.
4. The doctor took enough time for me.
5. The doctor gave me enough opportunities to state my difficulties and problems.
6. My problems and needs were understood and taken seriously by the doctor.
7. The doctor determined all treatment measures together with me.
8. The doctor left the final treatment decision to me.
9. The doctor encouraged me to involve my relatives in my treatment.
